# Effective smoking cessation interventions in people with cancer: A systematic review and meta-analysis of randomized controlled trials

**DOI:** 10.18332/tpc/211165

**Published:** 2025-12-19

**Authors:** Livingstone Aduse-Poku, Hui G. Cheng, Oxana Palesh, Susan Hong

**Affiliations:** 1 Massey Comprehensive Cancer Center, Virginia Commonwealth University, Richmond, United States; 2 Department of Psychiatry, Virginia Commonwealth University School of Medicine, Virginia Commonwealth University, Richmond, United States; 3 Division of Hematology, Oncology and Palliative Care, Department of Internal Medicine, Virginia Commonwealth University School of Medicine, Richmond, United States

**Keywords:** smoking cessation, cancer, interventions, randomized controlled trials, systematic review

## Abstract

**INTRODUCTION:**

Continued smoking increases the risk of all-cause and cancer-specific mortality and adversely affects treatment outcomes. We conducted a systematic review and meta-analysis of randomized controlled trials (RCTs) evaluating the effectiveness of smoking cessation interventions in individuals diagnosed with cancer.

**METHODS:**

We conducted a systematic review across several databases, including PsychINFO, EMBASE (through OVID), PubMed (also through OVID), and CINAHL, of RCTs evaluating smoking intervention trials of adult cancer survivors regardless of cancer type, stage, or treatment received. The Cochrane Risk of Bias Tool 2 (ROB-2) evaluated the risk of bias. A meta-analysis was performed using fixed-effect models in R. The last database search was conducted in March 2025.

**RESULTS:**

The search yielded 984 publications. Twenty-three studies met the inclusion criteria. Smoking cessation interventions significantly enhanced cessation rates compared to control (risk ratio, RR=1.36; 95% CI: 1.22–1.51). Results did not show a difference between behavioral interventions alone versus control in the random-effects model (OR=1.08; 95% CI: 0.74–1.60). For studies using biochemical verification, the RR was stronger (RR=1.57; 95% CI: 1.32–1.87) than studies using self-report (RR=1.25; 95% CI: 1.09–1.42). In studies reporting higher success rates in the intervention group, there was a high number of contacts and follow-ups, averaging at least five times.

**CONCLUSIONS:**

The present systematic review and meta-analysis provide robust evidence supporting the effectiveness of smoking cessation interventions in cancer patients, particularly when combining pharmacological and behavioral approaches. Cancer patients are capable of successfully quitting tobacco and should be encouraged by healthcare providers to initiate a smoking cessation effort that combines both approaches.

## INTRODUCTION

Smoking is a highly prevalent and preventable risk factor for many diseases and premature death^1^. Globally, there are more than 1.1 billion smokers and more than seven million smokers are killed by smoking-related adverse health effects each year^2^. According to the National Institute of Health (NIH), smoking is the leading cause of cancer and cancer-related deaths, accounting for nearly 10 million deaths annually in the United States^3^.

Cancer survivors who continue to smoke have an overall mortality rate that is 4% to 20% higher compared to those who had quit^4,5^. In addition, continued smoking after a cancer diagnosis increases the risk for adverse treatment outcomes, other chronic diseases such as COPD and cardiovascular diseases, risk for disease progression and second primary cancers, which significantly impairs cancer survivors’ quality of life^6,7^. Offering smoking cessation services is an important part of comprehensive cancer care treatment.

Individuals are more likely to discontinue smoking if the cancer is causally linked to their smoking behavior^8,9^. Despite harms associated with continued smoking, approximately 33% to 50% of cancer patients either continue to smoke or relapse after attempting to quit^10,11^. Factors contributing to this include poor cancer prognosis, increased stress for any reason, and co-existing anxiety and/or depression. In addition, the highly addictive nature of cigarette smoking makes the quitting journey a protracted process with high rates of relapse^12-14^.

Smoking cessation is the most effective way to mitigate smoking-related premature mortality and morbidity. Smoking cessation interventions can be classified into behavioral and pharmacological approaches. Pharmacological interventions include medications such as nicotine replacement therapy, varenicline, and bupropion^13,15^. Behavioral interventions encompass cognitive-behavioral therapy, motivational interviewing, telephone counseling, individual or group counseling, and web-based interventions^16^. Other approaches involve the combination of both pharmacological and behavioral interventions^17^. The combination of behavioral and pharmacological approaches are believed to work synergistically and enhance the likelihood of maintaining long-term abstinence.

Systematic reviews, comprising more than 600 studies, have been conducted to elucidate the most effective smoking cessation approaches for individuals without a history of cancer^18-23^. Yet, the generalizability of these studies to cancer survivors is unclear. A limited number of smoking cessation studies have specifically evaluated the effectiveness of these programs in the oncology population. Two systematic reviews that examined effective smoking cessation programs in cancer patients included observational studies and thus could not adequately evaluate the effectiveness of the smoking cessation interventions^24,25^.

A recent meta-analysis of observational studies and Randomized Controlled Trials (RCTs), found that a combination of pharmacological and behavioral interventions may be the most efficacious intervention for smoking cessation among cancer survivors^24^. This meta-analysis is of particular relevance because it was the first to evaluate the effectiveness of smoking cessation interventions in cancer patients based on experimental studies (e.g. RCTs) only, providing the highest quality of evidence for causal inference. This review addressed a critical research gap and offers tailored insights for smoking cessation among cancer survivors. Our results informed clinical practice, policymaking, and the future research needed to optimize care for cancer survivors, leading to advances in oncology care and public health.

## METHODS

A systematic review was performed in accordance with a predetermined protocol and was reported to be consistent with the Preferred Reporting Items for Systematic Reviews and Meta-Analyses (PRISMA) statement^26^. The protocol for this review was prospectively published as a preprint in medRxiv (https://www.medrxiv.org/content/10.1101/2025.01.11.25320383v1).

### Search strategy

The search strategy included primary keywords and specific vocabulary terms (see Supplementary file) for controlled searches of MeSH and Emtree across four databases. The search strategy involved four databases, including PsychInfo and EMBASE, both accessed through OVID as well as PubMed, available through OVID and the Cumulative Index to Nursing and Allied Health Literature known as CINAHL. The last search was conducted in March 2025. The full list of search terms and Boolean combinations is provided in the Supplementary file.

### Eligibility criteria

Eligibility criteria were specified according to the PICOS framework: Population (adult smokers diagnosed with cancer; Intervention (behavioral, pharmacological, or combined smoking cessation approaches); Comparison (no-treatment controls, usual care, or alternative interventions); Outcomes (smoking cessation assessed by self-report and/or biochemical verification); and Study design (randomized controlled trials, including cluster RCTs).

This review included studies which assessed smoking cessation methods for adult cancer survivors without considering the specific cancer type, stage or treatment history. The review included solely randomized controlled trials and cluster randomized controlled trials. The selected studies included no treatment controls along with usual practice and alternative intervention groups. Quasi-experimental trials with comparison groups, including non-randomized pre-post trials, time-series/interrupted time-series trials, preference trials, and regression discontinuity trials, were excluded (Supplementary file Table 1). Research articles published up to October 2024 were included. Only peer-reviewed articles in English were considered. The full list of search terms, keywords, Boolean operators, and filters applied is provided in the Supplementary file.

### Participants

The included studies involved adult participants diagnosed with any cancer, excluding non-melanoma skin cancer, who were current smokers. There were no limitations on cancer type, stage, or treatments.

### Types of interventions

Interventions were categorized into three groups including behavioral-only methods (like cognitive therapy and motivational interviewing), medication-only methods (like bupropion or varenicline and nicotine replacement therapy or their combination), and combined approaches which use both behavioral and medication treatments. Comparator groups in the included studies consisted of no-treatment controls, usual practice, or alternative interventions, as specified in the inclusion criteria.

### Outcome

The primary outcome was smoking cessation. Studies that measured smoking cessation quantitatively were selected for analysis. The study evaluated smoking cessation through point prevalence rates and continuous abstinence as well as current smoking status. Studies were included when they distinctly defined cessation through both self-reported abstinence and biochemical measures such as carbon monoxide in exhaled air or blood nicotine levels. The inability to establish any safe tobacco consumption level resulted in the exclusion of smoking reduction as an acceptable outcome. Effect measures used for synthesis were adjusted risk ratios (ARRs) with 95% confidence intervals (CI).

### Selection of studies and data extraction

Database searches were conducted by LAP. After removing duplicates, they were loaded into Covidence. Title and abstract screening were carried out independently by LAP and HC. LAP and HC independently reviewed all potentially eligible full-text articles. Disagreements were resolved through discussion. Extracted data included study characteristics, intervention details, participant numbers and attributes, outcomes, quit attempts, and follow-up duration. LAP and HC independently extracted data from the included studies. Covidence was used to automate duplicate removal and to manage screening and data extraction. Two reviewers (LAP, HC) worked independently at title/abstract and full-text stages; disagreements were resolved by consensus.

### Assessment of risk of bias in included studies

Risk of bias was assessed independently by two reviewers using the Cochrane Risk of Bias 2 (RoB-2) tool^27^, assessing five domains: 1) random sequence generation; 2) allocation concealment (for cluster trials, we considered the potential for selective recruitment or differential refusal based on known allocation, which could introduce ascertainment bias); 3) performance bias; 4) detection bias; and 5) attrition. We documented any other potential sources of bias not captured within these categories.

### Data analysis

The overall certainty of evidence for each outcome was assessed using the Grading of Recommendations Assessment, Development and Evaluation (GRADE) approach, considering risk of bias, inconsistency, indirectness, imprecision, and publication bias. Sensitivity analyses examined robustness to outcome verification method (biochemical vs self-report), intervention type (behavioral-only vs combined), and study risk of bias. We used summary tables and an initial narrative synthesis prior to quantitative pooling. Pooled estimates were calculated using fixed-effect models when between-study heterogeneity was low and random-effects models when heterogeneity was present; model choice was informed by chi-squared (χ^2^), I^2^, and τ^2^ statistics. Where necessary, data were standardized (e.g. harmonizing effect measures) prior to synthesis.

To evaluate outcomes, a meta-analysis was performed using fixed-effect models in R. We also applied random-effects models to assess differences in effect size estimates. The inverse variance method was used to calculate the risk ratio. To provide further insights, we stratified analysis by intervention type (behavioral interventions only and combined interventions) and by smoking cessation assessment (self-report only or with biochemical verification). A funnel plot was generated to assess potential publication bias. Asymmetry was evaluated visually and using the Egger test in R Studio. Covidence, an online systematic review management tool, was used to streamline screening, data extraction, and risk of bias assessment.

## RESULTS

### Study characteristics

**Figure 1:**
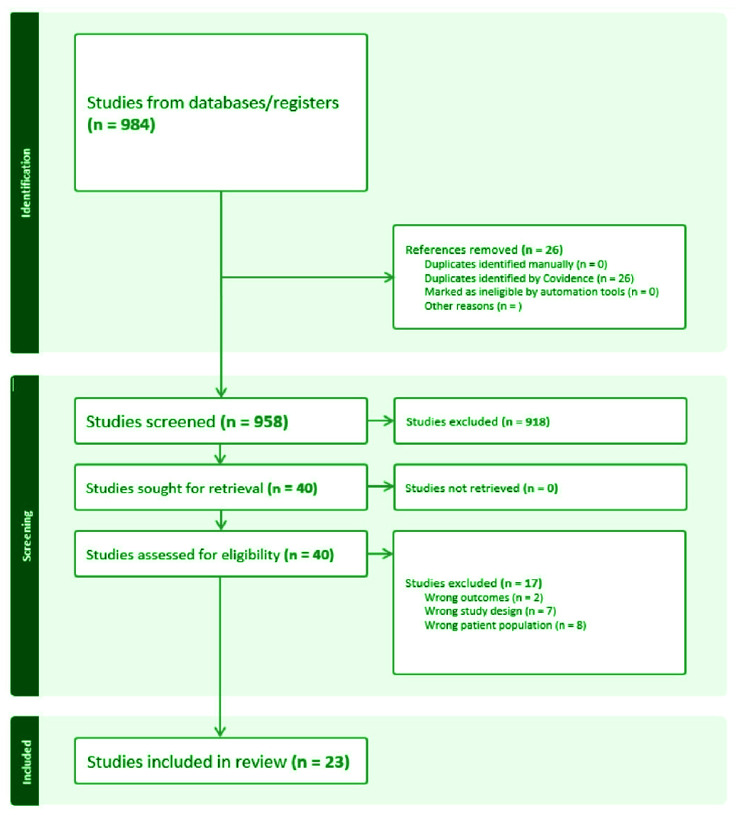
PRISMA flow diagram - Systematic review of effective smoking cessation in cancer patients

**Figure 2:**
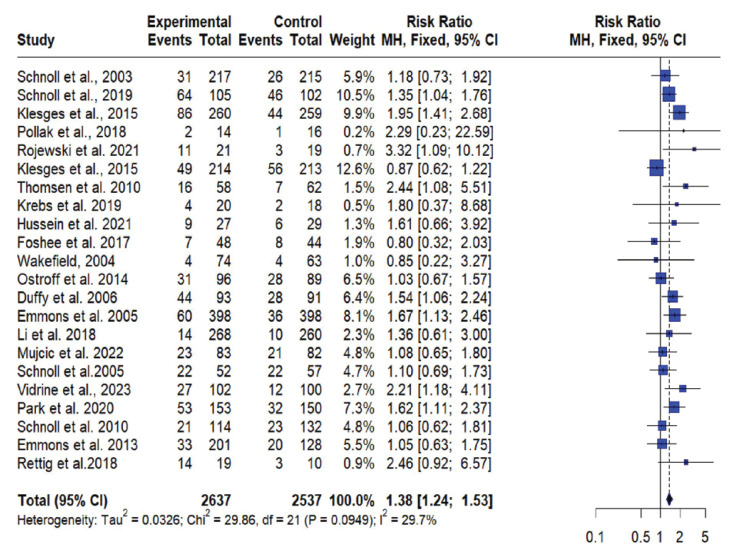
Forest plot of the efficacy of smoking cessation interventions in the experimental vs. control among cancer patients

The total number of participants was 5174 across 23 RCTs, with individual trial sample sizes varying substantially; interventions spanned behavioral, pharmacological, or combined formats, and follow-up durations ranged from several weeks up to 12 months. The average age of participants in the studies included in this review was 50.6 years. The search yielded 984 titles and abstracts. After removing duplicates and screening the titles and abstracts, full texts were retrieved for 40 potentially relevant studies (Figure 1). Twenty-three studies met the inclusion criteria and were included in the systematic review^27-49^. Of these 23 studies that met the eligibility criteria (Table 1) and were included in this review, 22 were included in the meta-analysis (Figure 2). The total sample size across all the studies was 5174 participants, and all studies were conducted between 2003 and 2023. The mean age of participants across all studies was 55 years. Most of the studies (17 of the 23, 73.9%) were conducted in the United States^28-44^. Other countries include Denmark, Lebanon, Australia, and the Netherlands, each contributing one study. Twelve studies were conducted across multiple centers^28,29,31,36,37,39-41,45-48^.

**Table 1:** Summary of study characteristics

*Authors Year*	*Study dates*	*Single or multicenter*	*Setting*	*Country*	*Aim*	*Inclusion criteria*	*Number of patients*	*Mean age (years)*	*Tumor site*
Schnoll et al.^28^ 2019	May 2013-June 2017	Multicenter	Two cancer centers	United States	Evaluate efficacy, safety, and adherence of 24-week varenicline for smoking cessation in cancer patients.	Age ≥18 years, diagnosis of cancer or recurrence within the past 5 years, smoking ≥5 cigarettes per week, and self-reported interest in quitting smoking.	207	58.5 (SD=9.4)	Genitourinary (25.6%), breast (22.0%), lung (17.1%), skin (11.0%), hematological (9.8%), head and neck (5.5%), gastrointestinal (4.9%), other
Schnoll et al.^45^ 2003	Multicenter	Multicenter	Conducted at various locations such as Fox Chase Cancer Center, Dana-Farber Cancer Institute, Duluth Clinic, Park Nicollet Health Services, and AMC Research Center	United States	To evaluate the efficacy of a physician-based smoking cessation treatment in cancer patients	Cancer patients who smoked before diagnosis	432	Median (range), 55 (20-81)	Stage I-II cancer (of any type) or stage III-IV breast, prostate, or testicular cancer or lymphoma
Klesges et al.^29^ 2015	Not specified	Multicenter	Cancer clinics	United States	Evaluate the efficacy of two tobacco quitlines for smoking cessation among childhood cancer survivors	Diagnosed with cancer before age 21, remission for ≥1 year, smoking regularly for ≥1 year	519	Not specified	All types of childhood cancers
Pollack et al.^30^ 2018	Single-center	Multicenter	Four clinics in Duke Cancer Network	United States	To test the feasibility, acceptability, and preliminary efficacy of a smoking cessation program paired with a pain management program for cancer survivors.	Cancer diagnosis within 5 years, life expectancy of at least 1 year, pain score ≥3 on a 10-point scale, smoked ≥100 cigarettes in lifetime, ≥5 cigarettes per day in prior 7 days, willing to quit, age ≥18, English speaking, not participating in other smoking cessation trials, cognitively able to consent.	30	57	Breast, colon, lung, and other cancers (anal, chronic lymphocytic leukemia, laryngeal, lymphoma, etc.)
Rojewski et al.^31^ 2021	2014-2017	Multicenter	Two cancer center-based tobacco treatment programs	United States	To evaluate a pilot preoperative contingency management (CM) intervention for smoking abstinence.	The information from the document has been extracted and presented in tabular format.	40	56.9	Any type of operative cancer
Klesges et al.^32^ 2015	2010-2013	Single-center	Cancer Treatment Facility	United States	Assess the efficacy of two tobacco quitlines in cancer survivors	Adult cancer survivors, 21+, regular smokers, English speakers, phone access, any cancer subtype	427	Not specified	Not specified
Thomsen et al.^46^ 2010	April 2006 – December 2007	Multicenter	Breast surgical departments	Denmark	To examine if a brief smoking cessation intervention before breast cancer surgery reduces postoperative complications and increases smoking cessation	Daily smoking women, aged 18+, sufficient language proficiency; excluded if high alcohol intake, substance abuse, psychiatric disease, pregnancy, or preoperative chemotherapy	120	Median 57.0	Breast
Krebs et al.^33^ 2019	Not specified	Single-center	Hospital-based intervention for cancer patients	United States	To examine feasibility, acceptability, and preliminary outcomes of an interactive game for smoking cessation among cancer patients.	English-speaking patients with recent cancer diagnosis, scheduled for surgery, reported smoking in the past 30 days, able to use a computer game.	24	57.1 years	32% lung, 24% gastrointestinal, other
Smaily et al.^47^ 2021	Multicenter	Three tertiary care hospitals		Lebanon	To evaluate the effectiveness of a brief smoking cessation intervention in head and neck cancer patients.	Patients with confirmed HNSCC, >18 years old, undergoing biopsy/surgery/radiation therapy, active or recent smokers (past 3 months), reachable by phone, excluding those with drug addiction, psychiatric illness, severe cardiovascular conditions, and other health constraints.	56	60.9	Laryngeal, lip and oral cavity, oropharyngeal
Foshee et al.^34^ 2017	May 2012 – September 2013	Single-center	Department of Otolaryngology, Thomas Jefferson University Hospital	United States	Evaluate the efficacy of ‘The Easy Way to Stop Smoking’ book for smoking cessation among patients with head and neck cancer.	Current smokers, age >18 years, attending the clinic for care, consenting to participate.	52	Not specified	Head and neck cancer
Wakefield et al.^50^ 2004	May 1999 to January 2001	Single-center	South Australian public hospital	Australia	To determine whether a motivational interviewing intervention increased successful smoking cessation attempts among cancer patients. Cell.	Cancer diagnosis, smoking tobacco more than weekly, ability to consent, prognosis exceeding 6 months, proximity for follow-up, and later expanded to include remote patients.	137	52.3	Mixed, including lung (12%), head and neck (17%), bladder (2%), breast (13%), prostate (9%), colon (10%), leukemia (10%), lymphoma (15%), testicle (4%), and others (8%).
Ostroff et al.^35^ 2014	Not specified	Single-center	Hospital-based, pre-surgical intervention	United States	Evaluate the efficacy of a pre-surgical cessation intervention for newly diagnosed cancer patients.	English-speaking adults with a localized solid mass likely to be cancer; awaiting surgical treatment; smoked at least 8 cigarettes/day.	185	55.9	Thoracic (30%), head and neck (9%), breast (12%), gyn (12%), urology (20%), other (17%)
Duffy et al.^36^ 2006	2000-2003	Multicenter	VA Hospitals and University of Michigan	United States	To test a tailored intervention addressing smoking, alcohol use, and depression among head and neck cancer patients.	Patients with head and neck cancer screening positive for smoking, alcohol use, or depression; >18 years; not pregnant.	184	57.0	Larynx, oropharynx/ hypopharynx, oral cavity/other
Emmons et al.^37^ 2005	May 1999-July 2000	Multicenter	Not specified	United States	To evaluate the impact of a peer-based telephone counseling intervention on smoking cessation among childhood cancer survivors.	Age ≥18 years, not in cancer treatment, mentally able to consent, reading/speaking English, current smokers identified via CCSS.	796	31.0	Leukemia, CNS malignancies, Hodgkin’s disease, non-Hodgkin’s lymphoma, kidney cancer, neuroblastoma, soft tissue sarcoma
Mujcic et al.^49^ 2022	Nov 2016-Sep 2019	Single-center	Online	Netherlands	Evaluate effectiveness, cost-effectiveness, and cost-utility of a digital smoking cessation intervention (MyCourse) compared to a noninteractive web-based information brochure for cancer survivors.	Aged ≥18 years, diagnosed with cancer in the past 10 years, internet access, smoked ≥5 cigarettes/day in the past week, and intent to quit smoking. Excluded: pregnancy, severe mental health issues, etc.	165	54.2	Breast (45.5%), lung (13.9%), uterus (11.5%), head and neck (10.9%), colon (6%), other (12.1%)
Li et al.^48^ 2018	September 2012 – March 2015	Multicenter	Outpatient oncology clinics	Hong Kong	To evaluate the effectiveness of a smoking cessation intervention using a risk communication approach for cancer patients.	(a) Smoked weekly in the past 6 months; (b) Diagnosed with cancer; (c) Any stage of cancer; (d) Aged 18+; (e) Cantonese-speaking; Excluded if cognitive/mental illness.	528	58.9 ± 12.3	Lung, colorectal, prostate/testicle, liver/bile duct, stomach/pancreas, kidney/bladder, etc.
Schnoll et al.^38^ 2005	Not specified (22 months duration)	Single-center		United States	Evaluate the impact of CBT tailored to cancer patients for smoking cessation, compared to general health education.	Head and neck or lung cancer patients; reported smoking within the last 30 days; English-speaking; ability to attend hospital sessions and follow-up via phone.	109	58.2	Head and neck (31%), lung (69%)
Vidrine et al.^39^ 2023	Feb 2017 - Aug 2021	Multicenter	Clinic in Oklahoma City and online	United States	Evaluate the long-term efficacy of Motivation And Problem Solving (MAPS) for smoking cessation in cervical intraepithelial neoplasia (CIN) or cervical cancer survivors.	1) Age ≥18, 2) English/Spanish speakers, 3) History of CIN or cervical cancer, 4) Reported smoking (≥100 lifetime cigarettes + smoking in past 30 days), 5) Working phone, 6) Valid address.	202	48	Cervical (CIN/Cancer)
Park et al.^40^ 2020	November 2013 – July 2017	Multicenter	Massachusetts General Hospital/Dana-Farber/Harvard Cancer Center and Memorial Sloan	United States	To determine the effectiveness of sustained telephone counseling and medication compared to shorter-term counseling and medication advice in quitting smoking for cancer patients.	Adults recently diagnosed with cancer, who smoked ≥1 cigarette within 30 days, spoke English or Spanish, and had specific cancer types (breast, lung, etc.)	303	58.3	Breast, gastrointestinal, genitourinary, gynecological, head and neck, lung, lymphoma, or melanoma cancers
Schnoll et al.^41^ 2010	1 October 2002 – 1 April 2008	Multicenter		United States	To examine the effects of bupropion among cancer patients with significant depression symptoms on smoking cessation	18 years or older, English-speaking, smoking at least 2 cigarettes/day, cancer diagnosis.	246	54.8	Head and neck, lung, breast, prostate, colorectal
Ghosh et al.^42^ 2016	Not specified	Single-center	Tertiary care hospital	United States	To evaluate the effects of financial incentives on smoking cessation in head and neck cancer patients.	Patients aged >18 years, actively smoking ≥5 cigarettes/day, undergoing or having completed treatment for head and neck cancer.	24	59-61	Head and neck region
Rettig et al.^43^ 2018	May 2014 – March 2015	Single-center	Johns Hopkins cancer treatment centers	United States	Evaluate a novel smoking cessation intervention’s feasibility and efficacy during radiation therapy in cancer patients.	Patients aged 18+, English-speaking, undergoing ≥5 weeks radiation for head, neck, or thoracic malignancy, smoked in the past 14 days.	29	55	Head and neck (52%), thoracic (48%)
Emmons et al.^44^ 2013	May 2014 – March 2015	Single-center	Johns Hopkins cancer treatment centers	United States	To evaluate the efficacy of a novel smoking cessation intervention among cancer patients undergoing radiation therapy.	Age ≥18, English-speaking, planned radiation therapy for ≥5 weeks, and cigarette use in the prior 14 days.	329	55	Head and neck (52%), thoracic (48%)

AMC: AMC Cancer Research Center (American Medical Center). HNSCC: Head and neck squamous cell carcinoma. GYN: gynecology/gynecological cancers. CNS: central nervous system. CCSS: Childhood Cancer Survivor Study. VA: U.S. Department of Veterans Affairs (Veterans Affairs)

### Interventions utilized

**Figure 3:**
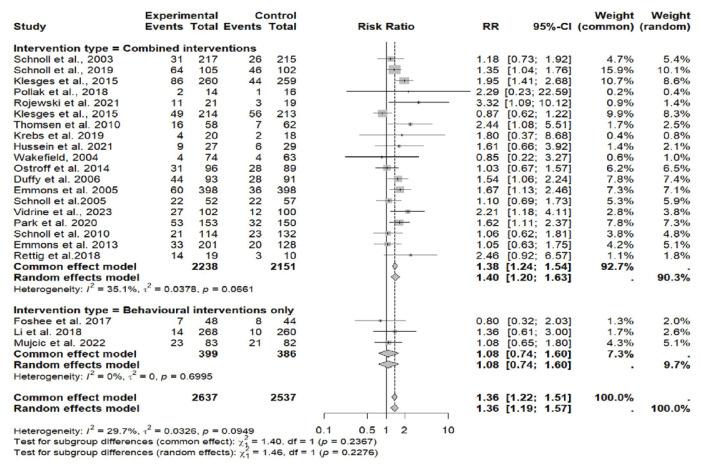
Forest plot of smoking cessation interventions: Risk ratios by intervention type (combined interventions vs. behavioral interventions only)

None of the studies included only pharmacological interventions; four studies included only behavioral interventions^34,42,48,49^, while 19 (82.6%) used a combination of behavioral and pharmacological interventions^28-31,34-46,48,49^ (Figure 3). Biochemical verification of abstinence was assessed via an exhaled carbon monoxide (CO) test or a cotinine test from urinary or saliva samples in 11 out of 22 studies^29,30,35,39-43,46,48,50^. Statistical heterogeneity was assessed using the Chi-squared (χ^2^) test, quantified with the I^2^ statistic, and by estimating between-study variance (τ^2^). Corresponding p-values were reported to evaluate the significance of heterogeneity.

*Behavioral interventions only*

None of the behavioral intervention trials showed a significant effect. Behavioral interventions in two studies included proactive and reactive quitline counseling, brief face-to-face advice with a booster session, and a digital smoking cessation program that combined motivational interviewing, cognitive-behavioral therapy (CBT), and acceptance and commitment therapy (ACT)^48,49^. Two studies provided the self-help book *The Easy Way to Stop Smoking* by Allen Carr^34^ for free, along with smoking cessation information and free enrollment in classes^42^ (Table 2).

**Table 2:** Summary of smoking cessation interventions used in the included studies

*Authors Year*	*Intervention*		*Control*		*Intervention tested*	
	*Pharmacological*	*Behavioral*	*Pharmacological*	*Behavioral*	*Pharmacological*	*Behavioral*
Schnoll et al.^28^ 2019	Nicotine replacement therapy (NRT)	Quit advice and assistance during a physician visit in accordance with the National Institutes of Health guidelines for physician-based smoking interventions	None	Usual care (standard physician advice)	Nicotine replacement therapy (NRT)	Quit advice and assistance during a physician visit in accordance with the National Institutes of Health guidelines for physician-based smoking interventions
Schnoll et al.^45^ 2003	24 weeks of varenicline (extended; ET)	Smoking cessation counseling (7 sessions over 24 weeks, 4 in-person and 3 by phone)	12 weeks of varenicline plus 12 weeks of placebo (standard; ST)	Smoking cessation counseling (7 sessions over 24 weeks, 4 in-person and 3 by phone)	Extended varenicline 24 weeks vs 12 weeks	None
Klesges et al.^29^ 2015	Proactive condition - were mailed a four-week supply of NRT in the form of the patch followed by another four weeks	Proactive (i.e. counselor-initiated calls)	2 weeks of NRT patch	Reactive (Participant-initiated intervention)	4 weeks vs 2 weeks of NRT	Proactive vs reactive
Pollack et al.^30^ 2018	Nicotine replacement therapy (NRT) patches	Smoking cessation and pain management counseling (4 weekly 60-min sessions via telephone)	Wait-list control		Nicotine replacement therapy (NRT) patches	Smoking cessation and pain management counseling (4 weekly 60-min sessions via telephone)
Rojewski et al.^31^ 2021	Nicotine replacement therapy (NRT) via nicotine patches	Counselling and monetary payment delivered contingent on abstinence	Nicotine replacement therapy (NRT) via nicotine patches	Standard care with monitoring only (MO), including counseling and nicotine replacement without no monetary incentives	None	Contingency management (i.e. monetary payment upon abstinence) vs no money payment
Klesges et al.^32^ 2015	Nicotine replacement therapy (NRT)	Proactive (i.e. counselor-initiated calls)	NRT and reactive (i.e. participant-initiated calls)	Participant-initiated calls	None	Counsellor initiated vs participant-initiated calls
Thomsen et al.^46^ 2010	Personalized nicotine replacement therapy provided free of charge, tailored to patient preferences and dependence	A one-session counseling of 45–90 min based on motivational interviewing principles, encouraging smoking cessation from two days before to ten days after surgery	None	Standard preoperative care, which included inconsistent or no advice about smoking risks and background/ objectives of the study	NRT	Motivational interviewing vs inconsistent or no advice
Krebs et al.^33^ 2019	Cessation pharmacotherapies offered as part of standard care (evidence-based behavioral and pharmacological practices)	QuitIT interactive coping skills game, a mobile app to help resist smoking urges using game design principles		Standard care: 4 telephone or bedside counseling sessions and print cessation educational materials	Smoking cessation pharmacotherapies	Mobile app vs 4 telephone or bedside counseling sessions and print cessation educational materials
Smaily et al.^47^ 2021	Nicotine patch therapy (NPT) for 8 weeks in gradually decreasing doses	Brief perioperative smoking cessation intervention using NIH 5As model, counseling, and resources	None	Usual care group (UCG): brief advice to quit smoking	Personalized nicotine replacement therapy	None
Foshee et al.^34^ 2017	None	Distribution of the self-help book *The Easy Way to Stop Smoking* by Allen Carr (provided for free)	None	Recommended to purchase *The Esay Way to Stop Smoking* book	None	Book provided for free vs Book recommended
Wakefield et al.^50^ 2004	Nicotine replacement therapy (NRT)	Motivational interviewing, booklets for cancer patients, advice for family to quit smoking	None	Brief advice to quit, generic brochures, state-based quit-line information	NRT	Motivational interviewing vs brief advice to quit
Ostroff et al.^35^ 2014	Nicotine replacement therapy (NRT)	Behavioral counseling with Scheduled Reduced Smoking (SRS) using a handheld device (QuitPal)	NRT	Standard best-practice tobacco cessation (counseling + NRT without SRS)	None	Behavioral counseling with Scheduled Reduced Smoking (SRS) vs Behavioral counseling without Scheduled Reduced Smoking (SRS)
Duffy et al.^36^ 2006	Nicotine replacement therapy (patch, gum, inhaler). bupropion. Selective serotonin reuptake inhibitors (paroxetine, fluoxetine, sertraline)	Cognitive Behavioral Therapy (CBT) workbook and 9–11 telephone counseling sessions. Tailored CBT focusing on smoking cessation, alcohol reduction, and mood management	None	Enhanced usual care, including assessment, counseling, and referrals to local resources	NRT, bupropion, and selective serotonin reuptake inhibitors	CBT vs usual counseling
Emmons et al.^37^ 2005	Nicotine replacement therapy (NRT), provided free to participants in the peer-counseling (PC) group.	Peer-delivered telephone counseling: up to six calls by trained survivors, targeted materials, motivational interviewing	None	Self-help: cessation manual (‘Clearing the Air’) and letter emphasizing quitting importance	NRT	Peer delivered telephone counselling vs self-help cessation manual
Li et al.^48^ 2018	None	Brief, face-to-face individualized advice based on risk communication with a booster session and smoking cessation booklet	None	Standard care with a self-help smoking cessation booklet	None	Face-to-face individualized advice and smoking cessation booklet vs standard care with a self-help smoking cessation booklet
Mujcic et al.^49^ 2022	None	MyCourse - Quit Smoking (digital interactive smoking cessation intervention using motivational interviewing, cognitive-behavioral therapy, and acceptance commitment therapy)	None	Non-interactive web-based information brochure on smoking cessation, accessible as usual care	None	Digital interactive smoking cessation intervention vs non-interactive web-based information brochure on smoking cessation
Schnoll et al.^38^ 2005	Nicotine replacement therapy (transdermal patch Nicoderm CQ^®^)	Cognitive-behavioral therapy (CBT) targeting psychological factors	NRT	General Health Education (GHE) and NRT	None	CBT vs general health education
Vidrine et al.^39^ 2023	Nicotine replacement therapy (patch + lozenge)	Motivation and Problem Solving (MAPS) counseling (6 sessions)	NRT	Standard treatment (ST) with referrals to tobacco quitline and self-help materials	None	Motivation and Problem Solving (MAPS) counseling vs standard treatment (ST) with referrals to tobacco quitline
Park et al.^40^ 2020	Provision of FDA-approved smoking cessation medications including nicotine replacement therapy, bupropion, or varenicline	Sustained telephone counseling with 4 weekly sessions, 4 biweekly sessions, and 3 monthly booster sessions	None	Shorter-term telephone counseling with 4 weekly sessions and advice regarding cessation medications	Provision of FDA-approved smoking cessation medications including nicotine replacement therapy, bupropion, or varenicline	Sustained telephone counseling with 4 weekly sessions vs short-term telephone counseling with 4 weekly sessions
Schnoll et al.^41^ 2010	Bupropion (sustained-release, 300 mg/day for 9 weeks)	Behavioral counseling (5 sessions)	Placebo, alongside NRT	Counseling	Bupropion vs placebo, alongside NRT	Behavioral counseling vs counseling
Ghosh et al.^42^ 2016	None	Information-only group: provided smoking cessation information, free enrollment in classes, and incentives in the form of cash payments at specific time intervals if class attendance or smoking abstinence was confirmed	None	Information-only group: provided smoking cessation information and free enrollment in classes	None	Incentives vs no incentives
Emmons et al.^44^ 2013	Free pharmacotherapy (nicotine patch or zyban) for participants and spouses	Web-based intervention (interactive, peer-moderated forum)	Free pharmacotherapy (nicotine patch or zyban) for participants and spouses	Print materials (tailored content, survivor testimonials) and free pharmacotherapy	None	Web based vs print materials
Rettig et al.^43^ 2018	Combination NRT (patch/gum, patch/lozenge, patch/nasal spray), bupropion, varenicline	Intensive counseling, motivational interviewing, text-messaging support, contingency management, educational materials, frequent follow-up	None	Enhanced usual care: single counseling session, workbook, cessation resources, mental health screening	Combination NRT (patch/gum, patch/lozenge, patch/nasal spray), bupropion, varenicline	Intensive counseling, motivational interviewing, text-messaging support vs enhanced usual care

AMC: AMC Cancer Research Center (American Medical Center). HNSCC: Head and neck squamous cell carcinoma. GYN: gynecology/gynecological cancers. CNS: central nervous system. CCSS: Childhood Cancer Survivor Study. VA: U.S. Department of Veterans Affairs (Veterans Affairs).

*Combined interventions*

Twenty studies reported the effects of smoking cessation interventions, combining both pharmacological and behavioral approaches. Five studies combined smoking cessation counseling, such as in-person, telephone-based, or group sessions, with NRT (patches, gum, inhalers)^28,30,45,46,50^. Other studies employed cognitive behavioral therapy (CBT) or motivational interviewing with pharmacological options such as NRT, bupropion, or varenicline^29,36,38,39,47,50^. Three studies used technology-based support, such as mobile apps and the web, with medications such as NRT and bupropion^33,35,37^. Two studies used contingency management (monetary rewards for smoking abstinence) or problem-solving-focused counseling with NRT or other smoking cessation medications^31,39^. Two studies used a mixture of counseling techniques, such as motivational interviewing, text messaging, frequent follow-ups, and educational materials, along with multiple pharmacological agents such as NRT, bupropion, and varenicline^40,43^. Also, studies included in this review also indicate that providing free medication was associated with a greater likelihood of quitting.

Eight of the studies showed a statistically significant effect^28,29,31,36,37,39,40,46^. Rojewski et al.^31^ combined cognitive behavioral tobacco treatment approaches and monetary payments contingent on CO-verified smoking abstinence with nicotine replacement therapy (NRT) using nicotine patches. Additionally, Vidrine et al.^39^ integrated motivation and problem-solving (MAPS) counseling, consisting of six sessions, with NRT via patches and lozenges. In all studies that reported a significantly higher success rate in the intervention group compared to the control, there was a higher number of contacts and follow-ups with participants, averaging at least five times^28,29,31,36,37,39,40^.

### Interventions tested

Intervention tested refers to difference in interventions between the experimental and control groups. In this review, 11 studies tested pharmacological and behavioral interventions, 10 tested only behavioral interventions, and two tested only pharmacological interventions (Table 2). Most studies that found a significant effect of smoking cessation interventions on quit rate provided different pharmacological interventions for both groups, primarily comparing nicotine replacement therapy (NRT) versus no NRT/placebo, different durations of NRT or other medication use, or a combination of medications versus no medication.

### Meta-analysis

**Figure 4:**
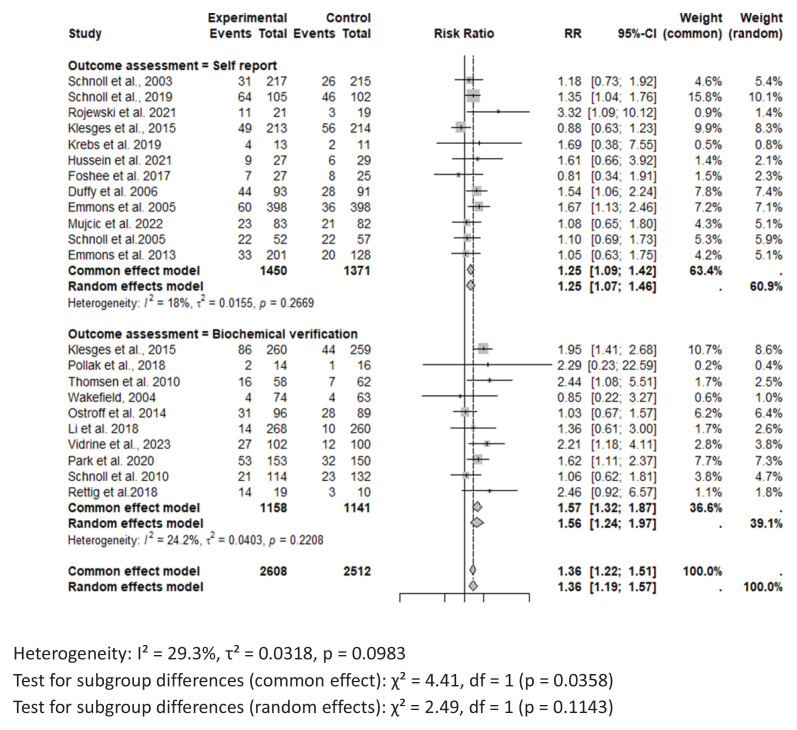
Forest plot of smoking cessation interventions: Risk ratios by outcome assessment method (self-report vs. biochemical verification)

For the meta-analysis, 22 RCTs comparing intervention to standard care were included. One study was excluded the from meta-analysis due to insufficient power^42^. Across all studies included in the meta-analysis, smoking cessation interventions significantly enhanced smoking cessation rates compared to standard care (RR=1.36; 95% CI: 1.22–1.51) (Figure 3). Results did not show a difference between behavioral interventions alone versus standard care in the fixed (common) effect model (OR=1.08; 95% CI: 0.74–1.60). A statistically higher smoking cessation rate was observed for combined (pharmacological and behavioral) interventions in the fixed (common) effect model (OR=1.38; 95% CI: 1.24–1.54) (Figure 4). For studies that used biochemical verification to assess smoking cessation, the RR was stronger (RR=1.57; 95% CI: 1.32–1.87) compared to studies that relied solely on self-report for smoking cessation assessment (RR=1.25; 95% CI: 1.09–1.42) (Figure 4).

Regarding the interventions tested (differences in interventions between the experimental and control groups), studies that implemented both pharmacological and behavioral interventions demonstrated a higher smoking cessation rate in the intervention group compared to the control (OR=1.50; 95% CI: 1.23–1.83). Similarly, studies focused solely on pharmacological interventions also showed a higher smoking cessation rate in the intervention group compared to the control (OR=1.37; 95% CI: 1.06–1.76). For studies that tested behavioral interventions exclusively, no significant difference in smoking cessation was observed between the intervention and control groups (OR=1.23; 95% CI: 0.98–1.54). The heterogeneity in these studies is low, as indicated by the non-significant χ^2^ test (I^2^=29.70%, p=0.0949), a modest χ^2^ value of 1.40, and a small τ^2^ value of 0.0318, suggesting that the differences in effect sizes across studies are likely attributable to random variation rather than systematic differences (Supplementary file Figure 1).

### Methodological quality assessment

Of the 23 randomized control trials, 4 were assessed to have a low risk of bias, 16 had a moderate risk of bias, and 3 had a high risk of bias (Supplementary file Figures 2 and 3). Most studies evaluated smoking cessation through self-reporting, leading to a rating of ‘serious’ for indirectness. However, the relatively small confidence interval of both the included and pooled studies resulted in a rating of ‘not serious’ for imprecision. Additionally, no significant publication bias was detected, as shown in the funnel plot and Egger’s test (z=1.0944, p=0.2738) (Supplementary file Figure 4). The overall quality of evidence was rated as moderate.

## DISCUSSION

This combined systematic review and meta-analysis is the first to evaluate the effectiveness of smoking cessation interventions in cancer patients based solely on experimental studies (e.g. RCTs), providing the highest quality evidence for causal inference. Eight out of the 23 studies included in this review demonstrated a significant effect of smoking cessation interventions on smoking cessation compared to controls. Our findings indicate that for individuals diagnosed with cancer, a combination of pharmacological and behavioral interventions offers the greatest likelihood of successful smoking cessation. These interventions result in a 36% higher success rate for quitting smoking compared to controls. In the intervention groups, 581 (22%) of 2637 patients quit, compared to only 399 (16%) of 2537 in the control groups. In all studies that reported a significantly higher success rate in the intervention group compared to the control, there was a high number of contacts and follow-ups with participants, averaging at least five times over a period of several weeks to months, suggesting the sustained engagement may be a key factor in successful cessation. Also, studies included in this review indicate that providing free medication was associated with a greater likelihood of quitting. Among the studies that employed behavioral interventions alone, the meta-analytic summary is marginally significant, reinforcing the importance of combination therapies in cessation strategies, particularly in medically vulnerable populations like cancer patients.

While most previous reviews have reported no significant differences in abstinence rates between the intervention arm and the control arm^51,52^, our review, which focused exclusively on RCTs and included more recent studies, identified a statistically significant difference between intervention and control groups. All studies with statistically significant results combined both pharmacological and behavioral interventions, with most testing both types of interventions. No statistically significant difference in quit rates was observed between intervention and control groups for studies that utilized only one type of intervention. The literature supports the effectiveness of these tobacco cessation interventions, as clinical practice guidelines recommend the use of combination therapies, which are associated with increased odds of achieving abstinence^53^. Combining pharmacological and behavioral interventions for smoking cessation is more effective than employing either approach alone, as it addresses both the physical dependence on nicotine and the psychological aspects of smoking behavior through counseling^54,55^. Moreover, medications help individuals focus on behavioral changes by alleviating withdrawal symptoms^56^. Additionally, those engaged in both medication and behavioral counseling are more likely to remain committed to quitting^57,58^. Studies included in this review also indicate that providing free medication is linked to improved treatment adherence, which in turn is associated with a greater likelihood of quitting^31,43^. This finding aligns with previous research, which has shown that removing financial barriers to accessing cessation medications can play a crucial role in helping individuals quit smoking^59,60^.

Though there was no single pattern for effective smoking cessation among cancer patients, the effective interventions tested both pharmacological and behavioral interventions in both groups. Most studies provided different patterns of pharmacological interventions for both groups, primarily comparing nicotine replacement therapy (NRT) versus no NRT/placebo, different durations of NRT or other medications, or a combination of medications versus no medication. They also tested behavioral interventions between the intervention and control groups. The most common behavioral interventions included sustained telephone counseling with four weekly sessions versus short-term telephone counseling with four weekly sessions^45^, peer-delivered telephone counseling versus a self-help cessation manual^40^, and behavioral counseling with scheduled reduced smoking (SRS) versus behavioral counseling without scheduled reduced smoking^39^. In all studies that reported a significantly higher success rate in the intervention group compared to the control, there was a high number of contacts and follow-ups with participants, averaging at least five times^28,29,31,36,37,39,40^. Similarly, an overview of systematic reviews found that consistent support and follow-up can play a crucial role in helping individuals quit smoking successfully^61^. One of the studies was judged to have insufficient power because of its relatively small sample size, which limited its ability to detect statistically significant differences in quit rates^42^.

In this study, we performed sensitivity analysis by method of verification of smoking cessation. Smoking cessation is usually verified through self-report or biochemical verification. Because it is known that self-reported abstinence can be inaccurate, biochemical verification is important^62,63^. In this review the studies that assessed smoking cessation via biochemical verification seemed to report stronger associations between smoking cessation interventions and quit rate compared to those that assessed smoking cessation using self-report only. Of the studies that used exhaled CO as a form of biochemical verification, most of them had a cut point of <10 ppm^40^, while others had a cutoff ranging from <4 ppm to <8 ppm^31,43,48^. According to the Society for Research on Nicotine and Tobacco workgroup, studies should utilize the standard CO cut point of <5–6 ppm in geographical areas with strong smoke-free legislation, low smoking prevalence, and relatively low levels of air pollution^63^. Another, biochemical verification method is measuring levels of cotinine, a nicotine metabolite found in the blood, saliva, and urine. In this review, the studies^29,39^ included used a cut-point of ≤20 mg/mL, which is below the recommended cut-point of <30–50 ng/mL for urine cotinine. Biochemical verification provides an objective measure to verify self-reported smoking status, which is essential for the accuracy and reliability of clinical trials^64^. Biochemical verification also helps in detecting relapse in individuals who might not report it, ensuring that interventions can be adjusted accordingly^64^.

Similar to previous reviews, we found that the studies used different but related interventions^65,66^. However, an I^2^ test indicated that the heterogeneity between studies was not statistically significant. This implies that the studies’ variability is likely due to chance rather than true differences. Also, we teased out the specific interventions that were tested and performed sensitivity analyses grouping together studies with similar intervention patterns. Additionally, we performed RoB and GRADE assessments which improve validity, reproducibility, and confidence in findings, supporting evidence-based public health policies and interventions.

### Limitations

That 12 out of 23 studies used self-reported abstinence as outcome measure may be considered a limitation. Self-report may be a suitable approach for measuring smoking abstinence in patient populations outside the general public, such as individuals with cancer, provided the study sample is sufficiently large. This method could potentially yield higher smoking cessation rates compared to biochemical verification. Also, participation bias may also influence the findings, as studies often only enrolled a fraction of eligible patients. It is possible that individuals with a preexisting motivation to quit smoking were more likely to participate, and their intrinsic motivation may have contributed to higher quit rates than would be expected in the general population of cancer patients. Additionally, 3 out of the 23 included studies had high risk of bias.

## CONCLUSIONS

This systematic review and meta-analysis provide robust evidence supporting the effectiveness of smoking cessation interventions in cancer patients, particularly when combining pharmacological and behavioral approaches. Our findings highlight the importance of sustained follow-up, support, and the removal of financial barriers to medication access in enhancing quit rates. Despite some studies exhibiting a high risk of bias, the overall quality of evidence remains moderate. Individuals diagnosed with cancer are capable of successfully quitting tobacco use and should be encouraged by their healthcare providers to initiate a smoking cessation effort. Given the importance of objective verification, future RCTs should incorporate standardized biochemical verification to enhance reliability and comparability of findings. Also, future research should focus on improving study designs by addressing selection bias and conducting long-term follow-up beyond 12 months.

## Supplementary Material



## Data Availability

The data supporting this research are available from the authors on reasonable request. Further data of the studies are given in the Supplementary file.
