# Supplementary material for: Effective smoking cessation interventions in people with cancer: A systematic review and meta-analysis of randomized controlled trials
Source: Tob Prev Cessat. 2025 Dec 19;11:10.18332/tpc/211165. doi: 10.18332/tpc/211165 (PMC12716162; doi:10.18332/tpc/211165)
Supplement: Supplementary file 1 [file TPC-11-59-s1.pdf]

SEARCH TERMS

For Medline and Embase

		Search terms
Patients diagnosed with cancer	1	neoplasm.mp. or exp Neoplasms/ neoplasm$.mp.
	2	exp Breast Neoplasms/ or cancer patients.mp.
	3	cancer patient$.mp.
	4	cancer survivor$.mp.
	5	cancer survivors.mp. or exp Cancer Survivors/
	6	exp Lung Neoplasms/ or lung cancer patients.mp.
	7	exp Colorectal Neoplasms/ or colorectal cancer survivors.mp.
	8	exp Prostatic Neoplasms/ or prostate cancer patients.mp.
	9	1 or 2 or 3 or 4 or 5 or 6 or 7 or 8
	10	Smoking Cessation.mp. or Smoking/ or exp Smoking Cessation/ or exp Smoking Prevention/
	11	Smoking Cessation programs.mp.
	12	Smoking Cessation program$.mp.
	13	Smoking Cessation Agent$.mp.
	14	Nicotine Replacement Therapy.mp.
	15	Bupropion.mp.
	16	Varenicline.mp. or exp Varenicline/
	17	Behavior Therapy.mp. or exp Behavior Therapy/

Smoking cessation interventions / program / methods	18	Motivational Interview$.mp.
	19	Self-Help Group$.mp.
	20	Cognitive Behavioral Therap$.mp.
	21	exp Hypnosis/ or hypnosis.mp.
	22	comprehensive counsel$.mp.
	23	10 or 11 or 12 or 13 or 14 or 15 or 16 or 17 or 18 or 19 or 20 or 21 or 22
Randomized controlled trials/ interventional studies	24	Randomized Controlled Trial.mp. or exp Randomized Controlled Trial/ or exp Double-Blind Method/ or Doubleblind.mp. or Single-Blind Method/ or Single-blind.mp. or Random Allocation.mp. or Random Allocation/ or Clinical Trials.mp. or exp Clinical Trial/
Combined	34	9 AND 23 AND 24

For PubMed and other databases

#1 "Neoplasms"[Mesh Terms] OR neoplasm*[tiab] OR cancer*[tiab] OR malginan*[tiab] OR oncology[tiab] OR tumor[tiab] OR tumour[tiab] OR carcinoma[tiab]

#2 "Tobacco Smoking"[Mesh] OR "Cigarette Smoking"[Mesh] OR "Cigar Smoking"[Mesh] OR "Smoking"[Mesh] OR tobacco[tiab] OR Smoke*[tiab] OR cigarette*[tiab] OR cigar[tiab] OR smoking[tiab] OR “nicotine dependence”[tiab]

#3 "Smoking Cessation"[Mesh] OR "Tobacco Use Cessation Devices"[Mesh] OR "Smoking Cessation Agents" [Pharmacological Action] OR "Smoking Cessation Agents"[Mesh] OR "Tobacco Use Cessation"[Mesh] OR quit*[tiab] OR stop*[tiab] OR Cessation[tiab] OR “Smoking Cessation”[tiab] OR “Tobacco Use Cessation Device*”[tiab] OR “Smoking Cessation Agent*”[tiab] OR “Tobacco Use Cessation”[tiab] OR “brief physician advice”[tiab] OR “physician advice”[tiab] OR “smoking cessation advice”[tiab] OR counseling[tiab] OR “cognitive behavioral therapy”[tiab] OR “nurse intervention”[tiab] OR “motivational interviewing”[tiab] OR “Stage-based intervention*”[tiab] OR “Print-based self-help”[tiab] OR “group therapy”[tiab] OR exercise*[tiab] OR “financial incentive”[tiab] OR “monetary incentive”[tiab] OR hypnotherapy[tiab] OR “nicotine replacement therapy”[tiab] OR bupropion[tiab] OR nortriptyline[tiab] OR varenicline[tiab] OR cystine[tiab] OR e-cigarette[tiab] OR antidepressant*[tiab] OR “tricyclic antidepressant*”[tiab] OR “monoamine oxidase inhibitor*”[tiab] OR “selective serotonin reuptake inhibitor*”[tiab] OR “atypical antidepressant*”[tiab] OR “St. John's wort”[tiab] OR anxiolytic*[tiab] OR buspirone[tiab] OR diazepam[tiab] OR doxepin[tiab] OR meprobamate[tiab] OR ondansetron[tiab] OR metoprolol[tiab] OR oxprenolol[tiab] OR propanolol[tiab] OR rimonabant[tiab] OR taranabant[tiab] OR clonidine[tiab] OR lobeline[tiab] OR dianicline[tiab] OR mecamylamine[tiab] OR nicobrevin[tiab] OR “nicotine vaccine*”[tiab] OR naltrexone[tiab] OR naloxone[tiab] OR buprenorphine[tiab] OR “silver acetate”[tiab]

#4 #1 and #2 and #3

Supplementary Figure 1. Forest plot of smoking cessation interventions: risk ratios by intervention test (combine vs pharmacological vs behavioral)

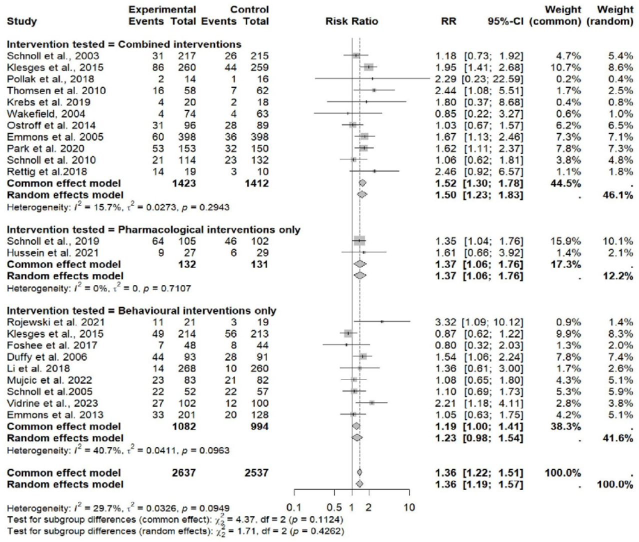

Supplementary Figure 2. Risk of bias across selected studies

Risk of bias domains

Study	D1	D2	D3	D4	D5	Overall
Schnoll et al., 2003	-	+	+	-	+	-
Schnoll et al., 2019	+	+	-	+	+	-
Klesges et al., 2015a	-	+	+	+	+	-
Pollak et al., 2018	-	-	+	-	+	-
Rojewski et al., 2021	+	+	-	+	+	-
Klesges et al., 2015b	+	-	+	+	+	-
Thomsen et al., 2010	-	+	+	-	+	-
Krebs et al., 2019	-	-	-	-	-	-
Hussein et al., 2021	+	-	+	-	+	-
Foshee et al., 2017	-	-	X	-	-	X
Wakefield et al., 2004	-	-	-	-	+	-
Ostroff et al., 2014	+	+	+	+	+	+
Duffy et al., 2006	-	-	X	+	+	X
Emmons et al., 2005	-	-	-	-	-	-
Li et al., 2018	+	-	+	-	+	-
Mujcic et al., 2022	+	-	-	X	+	-
Schnoll et al., 2005	-	-	+	X	+	-
Vidrine et al., 2023	+	-	+	-	+	-
Park et al., 2020	+	-	+	-	+	-
Schnoll et al., 2010	+	+	+	+	+	+
Ghosh et al., 2016	+	X	X	+	-	X
Emmons et al., 2013	+	+	+	+	+	+
Rettig et al., 2018	+	+	+	+	+	+

Domains:
D1: Bias arising from the randomization process.
D2: Bias due to deviations from intended intervention.
D3: Bias due to missing outcome data.
D4: Bias in measurement of the outcome.
D5: Bias in selection of the reported result.

Judgement
X High
- Some concerns
+ Low

Supplementary Figure 3. Summary of risk bias across key domains in included studies

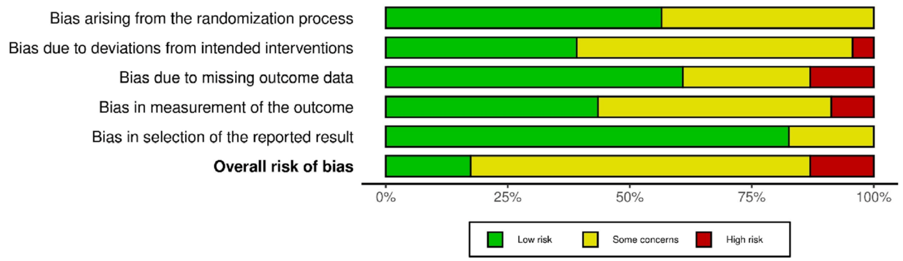

Supplementary Figure 4. Funnel plot to test for publication bias

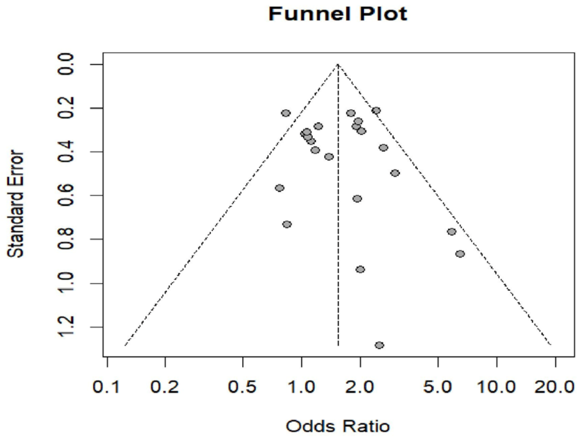

Regression Test for Funnel Plot Asymmetry
Model: mixed-effects meta-regression model
Predictor: standard error
Test for Funnel Plot Asymmetry: z = 1.0944, p = 0.2738
Limit Estimate (as sei -> 0): b = 0.1960 (CI: -0.2704, 0.6624)

Supplementary Table 1. Excluded studies and their reasons for exclusion

Study, year	Study title	Primary reason for exclusion
Bade, 2016	Effect of smoking cessation counseling within a randomized study on early detection of lung cancer in Germany	Wrong study design
Bastian, 2013	Assessment of the impact of actively proactive telephone counseling to promote smoking cessation among cancer patients and networks	Wrong patient population
Brain, 2017	Impact of low-dose CT screening on smoking cessation among high-risk participants in the UK Lung Cancer Screening Trial	Wrong patient population
Carroll, 2018	Cancer-related disease factors and smoking cessation treatment: Analysis of an ongoing clinical trial	Wrong study design
Cottrell-Daniels, 2024	Smoking Cessation by Cancer Treatment Status Among Cervical Cancer Survivors	Wrong study design
Crawford, 2019	Predictors of Varenicline Adherence Among Cancer Patients Treated for Tobacco Dependence and Its Association With Smoking Cessation	Wrong study design
Croghan, 2003	Comparison of nicotine patch alone versus nicotine nasal spray alone versus a combination for treating smokers: a minimal intervention, randomized multicenter trial in a non-specialist setting	Wrong patient population

Kanera, 2016	Use and Appreciation of a Tailored Self-Management eHealth intervention for Early Cancer Survivors: Process Evaluation of a Randomized Controlled Trial	Wrong outcomes
McAlister, 2004	Telephone assistance for smoking cessation: one year cost effectiveness estimations	Wrong patient population
McNaughton, 2013	Extended interactive voice response telephony (IVR) for relapse prevention after smoking cessation using varenicline and NRT: a pilot study	Wrong outcomes
Park, 2015	Primary Care Provider-Delivered Smoking Cessation Interventions and Smoking Cessation Among Participants in the National Lung Screening Trial	Wrong study design
Park, 2011	A smoking cessation intervention for thoracic surgery and oncology clinics: a pilot trial	Wrong patient population
Park, 2006	A process evaluation of a telephone-based peer-delivered smoking cessation intervention for adult survivors of childhood cancer: the partnership for health study	Wrong study design
Pike, 2007	American Cancer Society's QuitLink: randomized trial of internet assistance	Wrong patient population
Santi, 2022	Rates of Smoking Cessation at 6 and 12 Months after a Clinical Tobacco Smoking Cessation Intervention in Head and Neck Cancer Patients in Northern Ontario, Canada	Wrong study design
Sterba, 2011	Dyadic efficacy for smoking cessation: preliminary assessment of a new instrument	Wrong patient population
Strecher, 2005	A randomized controlled trial of multiple tailored messages for smoking cessation among smokers	Wrong patient population

Supplementary Table 2. Summary of outcome and results in the included studies

Author, year	Number of patients in the intervention groups	Number of patients in the control group	Outcome assessment	Primary outcome	Results
Schnoll et al., 2003	217	215	Self-reported smoking status	7-day point prevalence abstinence at 6 and 12 months	No significant difference in quit rates at 6 months (14.4% intervention vs. 11.9% control) or 12 months (13.3% intervention vs. 13.6% control).
Schnoll et al., 2019	105	102	Self-reported abstinence verified by carbon monoxide breath sample (<10 ppm)	7-day point prevalence and continuous abstinence at weeks 24 and 52	Adherence influenced better outcomes at week 24 for ET adherent patients (60.5% vs. 44.7%).
Klesges et al., 2015	260	259	Cotinine levels measured using NicAlertTM (cutoff <10 ng/ml)	Cotinine-verified smoking cessation at 12 months	Participants randomized to the Proactive + 4 weeks of medication condition self-reported a higher rate of cessation than those survivors in the Reactive + 2 weeks of medication condition at 8 weeks (33.2% vs . 17.0%, p < .001), but cessation rates were not significantly different at 12 months (23.0% vs. 18.7%, p = .29).
Pollak et al., 2018	14	16	Saliva cotinine testing for abstinence confirmation	Saliva-confirmed 7-day point prevalence abstinence	14% cessation in intervention arm vs. 6% in control arm at 2 months. Survivors in intervention reported less pain, improved depressive symptoms, and better physical function.
Rojewski et al. 2021	21	19	Seven-day point prevalence abstinence	Seven-day point prevalence abstinence	End of treatment: 52% abstinent (CM) vs. 16% (MO) (Risk Ratio = 3.2, p = .03). 3-month

			at end of treatment (day of surgery) and 3-month follow-up	at end of treatment (day of surgery) and 3-month follow-up	follow-up: 43% abstinent (CM) vs. 5% (MO) (Risk Ratio = 8.4, p = .02)
Klesges et al., 2015	214	213	Cotinine-verified smoking cessation at 12-month follow-up.	Self-reported point prevalence abstinence at 12 months: 22% (Proactive) vs. 26% (Reactive), but 48% of these failed biochemical verification.	
Thomsen et al. 2010	58	62	Perioperative smoking cessation and long-term smoking cessation self-reported with biochemical confirmation (exhaled CO levels).	Perioperative smoking cessation and long-term smoking cessation self-reported with biochemical confirmation (exhaled CO levels).	Perioperative smoking cessation: Modest improvement in the intervention group (28% vs. 11%, RR 2.49, 95% CI 1.10–5.60). Long-term smoking cessation at 12 months: No significant difference (13% intervention vs. 9% control, RR 1.48, 95% CI 0.50–4.38).
Krebs et al. 2019	20	18	Smoking abstinence and self-confidence to quit	Smoking abstinence and self-confidence to quit	Abstinence rates: QuitIT: 4/13 (30%), SC: 2/11 (18%)
Hussein et al. 2021	27	29	Smoking cessation rates at 3, 6, and 12 months (7-day point-prevalence of abstinence)	Smoking cessation rates at 3, 6, and 12 months	At 3 months, 57.1% (UCG) vs. 57.7% (SIG), p = 0.96. At 6 months, 42.9% (UCG) vs. 24% (SIG), p = 0.148. At 12 months, 33.3% (UCG) vs. 20.8% (SIG), p = 0.318
Foshee et al. 2017	48	44	Smoking cessation rates at short-term (2 weeks to 6 months) and long-term (6 months to 1 year) intervals.	Smoking cessation (defined as the participant reporting that they had quit smoking).	Smoking cessation rates: 26% in the free book group vs. 32% in the recommended purchase group (p = 0.76). Reading the book did not significantly correlate with cessation (p = 0.81).

Wakefield, 2004	74	63	Smoking cessation rates, biochemically confirmed	Smoking cessation rates, biochemically confirmed	3-month quit prevalence: Intervention 5%, Control 6%; Sensitivity analysis: Intervention 29%, Control 18% (p=0.32)
Ostroff et al. 2014	96	89	Primary: 7-day point prevalence abstinence at 6 months, verified by saliva cotinine levels.	7-day point prevalence abstinence at 6 months.	Abstinence at 6 months: BP-only: 32%; BP+SRS: 32%. Significant reductions in cigarette consumption for both groups; no effect on long-term abstinence rates.
Duffy et al. 2006	93	91	Smoking cessation rates	Smoking cessation rates	Smoking cessation: 47% in intervention group vs. 31% in control group (p < 0.05).
Emmons et al. 2005	398	398	7-day point-prevalence smoking status at 8 and 12 months.	Smoking cessation rates.	8-month quit rate: 16.8% (PC) vs. 8.5% (SH), p=0.0003. 12-month quit rate: 15% (PC) vs. 9% (SH), p=0.01.
Li et al. 2018	268	260	Self-reported 7-day point-prevalence smoking abstinence at 6 months. Biochemically validated quit rate at 6 months	Self-reported 7-day point-prevalence smoking abstinence at 6 months. Biochemically validated quit rate at 6 months	15.7% (intervention) vs. 16.5% (control), OR 0.94, 95% CI 0.59–1.50. Biochemically validated quit rate at 6 months (5.2% ((intervention) vs. 3.8% (control), OR 1.38, 95% CI 0.60–3.16)
Mujcic et al. 2022	83	82	Self-reported 7-day smoking abstinence at 6-month follow-up (primary outcome);	Self-reported 7-day smoking abstinence at 6 months	At 6 months, quit rates were 28% in MyCourse group and 26% in control group (no significant difference). MyCourse participants showed a greater reduction in cigarettes smoked at 12 months, but MyCourse was associated with higher societal costs.
Schnoll et al.2005	52	57	30-day point-prevalence abstinence	Smoking cessation (self-reported	1-Month Abstinence: CBT: 44.9%, GHE: 47.3%. 3-Month Abstinence: CBT: 43.2%, GHE: 39.2%.

				abstinence in the last 30 days)	No significant difference between CBT and GHE at both time points
Vidrine et al., 2023	102	100	Self-reported and biochemically confirmed smoking abstinence	Self-reported 7-day point prevalence abstinence from smoking at 18 months	MAPS group had significantly higher abstinence rates at 12 months (26.4% vs. 11.9%; p=0.017); no significant difference at 18 months (14.2% vs. 12.9%; p=0.79)
Park et al. 2020	153	150	Biochemically confirmed 7-day point prevalence tobacco abstinence at 6 months	Biochemically confirmed 7-day point prevalence tobacco abstinence at 6 months	Abstinence at 6 months was 34.5% in the intensive treatment group and 21.5% in the standard treatment group (difference, 13.0%; OR, 1.92; 95% CI, 1.13-3.27; p < 0.02)
Schnoll et al. 2010	114	132	7-day point-prevalence abstinence at Week 12 (end of treatment) and Week 27 (6 months post quit date), confirmed by CO levels.	7-day point-prevalence abstinence at Week 12 (end of treatment) and Week 27 (6 months post quit date), confirmed by CO levels.	At Week 12, the abstinence rates were 24.2% for placebo and 27.2% for bupropion; at Week 27, abstinence rates were 17.4% for placebo and 18.4% for bupropion.
Ghosh et al. 2016	6	8	Biochemical verification: exhaled carbon monoxide and urine cotinine tests at 30 days, 3 months, and 6 months	Smoking cessation sustained for six months	Only 2 participants quit smoking for six months, both from the incentive group. Financial incentives were deemed largely ineffective.
Emmons et al. 2013	201	128	Smoking cessation (self-reported 30-day abstinence at 15 months)	Smoking cessation (self-reported 30-day abstinence at 15 months)	Smoking cessation rates: Web: 16.5% (22/132). Print: 15.5% (20/127)
Rettig et al.2018	19	10	7-day smoking abstinence at 8 weeks	7-day smoking abstinence at 8 weeks	Smoking abstinence at 8 weeks: Intervention (74%) vs. Control (30%) (p=0.046)

			confirmed by exhaled CO (≤8 ppm)	confirmed by exhaled CO (≤8 ppm)	

Supplementary Table 3. Summary of findings: Pharmacological and behavioral interventions compared to control for smoking cessation

Pharmacological and behavioral interventions compared to control for smoking cessation

Patient or population: Cancer patients

Intervention: pharmacological and behavioral interventions

Comparison: control

Outcomes	**Anticipated absolute effects*** (95% CI)		Relative effect (95% CI)	№ of participants (studies)	Certainty of the evidence (GRADE)	Comments
	**Risk with control**	**Risk with pharmacological and behavioral interventions**				
Smoking Cessation assessed with: Self report or biochemical verification follow-up: range 3 to 12	157 per 1,000	**214 per 1,000** (187 to 247)	**RR 1.36** (1.19 to 1.57)	5174 (23 RCTs)	⨁⨁⨁◯ Moderate	

***The risk in the intervention group** (and its 95% confidence interval) is based on the assumed risk in the comparison group and the **relative effect** of the intervention (and its 95% CI).

CI: confidence interval; **RR:** risk ratio

GRADE Working Group grades of evidence
High certainty: we are very confident that the true effect lies close to that of the estimate of the effect.
Moderate certainty: we are moderately confident in the effect estimate: the true effect is likely to be close to the estimate of the effect, but there is a possibility that it is substantially different.
Low certainty: our confidence in the effect estimate is limited: the true effect may be substantially different from the estimate of the effect.
Very low certainty: we have very little confidence in the effect estimate: the true effect is likely to be substantially different from the estimate of effect.

© 2025 Aduse-Poku L. et al.
